# Influence of different host blood meal sources on the reproductive outcomes in *Anopheles gambiae*: Enhancing fecundity in a mass rearing environment

**DOI:** 10.1371/journal.pone.0307789

**Published:** 2025-03-10

**Authors:** Celestine N. Wekesa, Maxwell G. Machani, Nabie M. Bayoh, Z. Ngalo Otieno-Ayayo, Maurice V. Ombok, Eric O. Ochomo

**Affiliations:** 1 Kenya Medical Research Institute, Centre for Global Health Research, Kisumu, Kenya; 2 President’s Malaria Initiative Kinga Malaria, Kisumu, Kenya; 3 President’s Malaria Initiative Evolve Project, Lusaka, Zambia; 4 Department of Physical, Biological and Health Sciences, Rongo University, Rongo, Kenya; Manonmaniam Sundaranar University, INDIA

## Abstract

Identification of blood sources for maximum production of *Anopheles* mosquitoes is an important consideration for colony maintenance which involves mass rearing. High feeding rates, eggs production, hatching rates, larval, pupal, and adult survivorship are essential parameters to consider when selecting a blood host for mass production of *An. gambiae.* Here, we investigated the feeding success, reproduction performance and survivorship of *An. gambiae* when fed on blood from five different hosts: cow, goat, sheep, pig, and chicken compared to human blood. There was significant variations in feeding success (F_5, 18_ =  35.34, *p* < .001), egg laying (F _5,18_ = 12.57, *p* < .001), number of eggs laid (F_5, 18_ = 34.23 *p* < .001), egg hatchability (F _5, 114_ =  37.63, *p* < .001), pupation time (F _5,18_ =  5.532763, *p* = 0.0029) and pupation rates (F _5,18_ =  8.26, *p* < .001). Feeding success was highest in human blood meal (Mean = 125.25 ±  3.86), followed by pig blood meal (Mean = 123 ±  7.93), with no statistically significant difference between the two. The highest proportion of females that laid eggs were those fed on human blood (Mean =  36.50 ±  2.08) followed by those fed on chicken blood meals (Mean =  27.50 ±  5.44) and then pig blood meal (Mean =  26.25 ±  2.87). The mean number of eggs laid per mosquito was highest among those fed on human blood meal (111.65 ±  5.74) followed by those fed on pig blood meal (100.46 ±  6.36). The most favorable outcomes were observed with human blood for hatchability (88.35 ±  5.61%), pig blood for pupation percentage (Mean =  83.50 ±  4.79), and goat blood for pupation time (9.79 ±  0.41 days). Larval survival rates did not significantly differ among blood meal sources (F _5,92_ =  0.13, *p = *0.985). Nonetheless, the highest survival rate was observed with pig blood meal (Mean =  0.57 ±  0.11). Survival rates of adult F_1_ varied significantly across blood meals (*F*_6,346_ = 133.19, *p* < .001), with human blood meal showing the highest survival rate (Mean =  0.73 ±  0.04). However, pig blood meal (56 days) demonstrated the longest survival period, close to human blood meal (57 days). This study revealed pig blood as an excellent alternative to human blood meal for the mass production of *An. gambiae.*

## Background

The primary vector responsible for malaria transmission in sub-Saharan Africa, *Anopheles gambiae*, relies majorly on blood for the development of eggs for reproduction purposes [1]. The quality and type of bloodmeal host remain a significant factor in determining fecundity and reproduction success [2]. Proper knowledge of the influence of different host bloodmeal sources on reproductive outcomes of *Anopheles gambiae* is crucial in optimizing the *Anopheles* mass rearing programs, particularly for malaria control initiatives. Strategic selection of blood source for mass rearing enhances fecundity and improves the efficiency in production and maintenance of vector control programs in a mass rearing environment. The research areas that require mass rearing of mosquitoes include genetic manipulations, biological and physiological characterization, vector taxonomy and ecology, exploring novel bioactive agents, insecticides, and repellents against mosquitoes [3]. Additionally, the mass release of genetically modified mosquitoes is proposed as a preventive measure to halt the spread of the parasite as stated in the first supporting element [4]. Vector control approaches exploiting genetic modification have a huge demand for mass rearing of mosquitoes and are often faced with the challenge of sustainable non-human blood meal sources to sustain egg production [4]. A similar challenge is faced by research insectaries producing large numbers of mosquitoes for different experiments given their importance as disease vectors of numerous pathogens and parasites which include filariae, arboviruses and protozoans that cause human diseases [5].

Novel tools such as next generation vector control strategies, is one of the broad areas still under investigation that require mass rearing to meet the demands of the huge numbers needed for experimentation. In the advent of innovations aimed at curbing mosquito populations and mitigating pathogen transmission, various techniques have emerged such as the evolvement of new intervention strategies like spatial repellents and attractive sugar baits/attractive targeted sugar baits (ASB/ATSB) which require mass numbers of mosquitoes for testing their efficacy. With the assessment of novel prequalified formulations and chemistries such as Interceptor G2, Olyset plus and Permanent 3.0 for insecticide treated nets and indoor residual spraying products like broflanilide, several evaluations on these products demand enormous supply of laboratory reared mosquitoes for preliminary bio-efficacy testing [6]. The next generation vector control strategies such as Clustered regulatory interspaced short palindromic repeats (CRISPR) and the CRISPR associated gene Cas9 are the most current frontiers in precise insect genome mediated editing systems [7]. Other methods include precision-guided sterile insect technique [8], Gamma-Radiated Sterile Insect Technology [9], Dominant lethal gene [10], Female-Specific RIDL (fsRIDL) [11], symbionts for blocking malaria pathogen transmission, and Incompatible Insect Technology [12]. Successful genetic engineering of self-limiting OX513A strain of *Ae. aegypti* in Brazil [13,14] and field trial of genetically modified *Anopheles stephensi* in Djibouti both require mass production of mosquitoes to meet release demands [15]. Each of these cutting-edge strategies for vector control necessitates the extensive production of mosquitoes within an insectary before releasing genetically modified individuals or eggs into wild populations.

To establish high production of *Anopheles* mosquitoes that are sufficiently fit for their intended use to support these genome-based control approaches, blood meal sources that results in high number of eggs and high hatching rates is an essential factor [16]. When rearing mosquitoes, one challenge is determining the optimal source of blood [4] that result in successful mass production and subsequent supply of mosquitoes for downstream experiments and colony maintenance [17]. Anautogenous female mosquitoes must take a blood meal for protein to complete oogenesis, which is characterized by the development of the ovarian tissues resulting in the development of eggs [18]. *Anopheles* mosquitoes are likely to feed on humans and a range of animals including cattle, horses, goats, sheep, pigs, dogs, cats, rabbits, chicken and ducks [19]. Prior studies have indicated the significant impact of blood meal sources on feeding success, fecundity, hatchability, and pupation time for *Anopheles arabiensis* and *An. gambiae sensu stricto* [4,20]. In contrast, Mamai found no significant effects of host blood meal sources on egg production in mass rearing, particularly when comparing pig and cow blood sources [3]. Several studies have highlighted that *Anopheles* mosquitoes tend to achieve feeding success and varied reproductive outcomes when provided with blood from diverse sources. For instance, Gunathilaka *et al* [21] demonstrated that the choice of blood meal source significantly influences feeding rates and reproductive parameters in laboratory conditions for *Aedes aegypti*. Similar effects were observed in *Aedes* and *Anopheles* species, with different blood meal sources impacting feeding rates, adult survival, fecundity, hatching rates, and mosquito longevity [17]. However, most studies focus on parameters like blood meal quantity or preference, and often lack a comprehensive analysis involving multiple blood sources. Thus, this study was designed to assess the impact of six host blood sources (human, cow, goat, sheep, pig, and chicken) on the feeding success, reproductive performance, and survivorship of *An. gambiae* mosquitoes, with the aim of informing mass rearing and production practices.

## Materials and Methods

### Mosquito test population used and rearing conditions

The study was carried out at Kenya Medical Research Institute, Centre for Global Health Research insectaries in Kisumu, western Kenya in 2022. Laboratory test population of *An*. *gambiae* s.s*.* Kisumu strain was used. Rearing conditions in the insectary were maintained at 28ºC ±  5 and humidity of 80% ± 10 being controlled by an electric heater and humidifier. The adult mosquitoes were reared in 60x60x60 cm cages and were provided with 10% sugar solution that was soaked in cotton wool and placed on the cages. A photoperiod of light and dark cycles (12:12) was maintained.

### Blood-meal collection and preservation

The selection of host blood for this study was based on the availability of animal hosts in the study area in western Kenya and blood meal source studies that have reported mosquitoes feeding on multiple hosts [22,23]. The blood samples (Table in S1 Table) were collected from local abattoirs around the study area in Kisumu city located at an altitude of 0.091702 and a latitude 34.767956. Each host blood was collected in well-labeled sterile 50ml Falcon tubes containing 15mg of EDTA to attain a concentration of 0.3g/dm^3^ to prevent collected blood from clotting [24]. The collected blood was placed in a cool box and transported to the KEMRI laboratories for blood feeding. Screened human blood samples that were not infectious but unsuitable for transfusion (surplus in demand, underweight and quality issues due to storage) were collected from the local blood bank. Blood was stored at 4ºC until ready for use for a period of 14 days.

### Blood preparation and membrane feeding (Hemotek)

Three days old female *An. gambiae* s.s. mosquitoes starved for 12 hours were transferred to well labeled paper cups for blood feeding using hand-held aspirator. Blood meal was removed from the refrigerator 30 minutes prior and about 3ml was added to the Hemotek blood Membrane feeder machine (PS-6 System, Discovery Workshops, Accrington, UK) and fastened with a parafilm. Mosquitoes were allowed to feed for 30 minutes, followed by a 1-hour resting period to reduce stress, mortality and enhance physical recovery before being sorted into feeding status. Blood feeding per host blood meal was done in four replicates of 140 mosquitoes each. The feeding success defined as the mean number of mosquitoes that ingested blood meal, was assessed by examining the engorgement of the abdomens and categorized as either fully fed, half fed or unfed. Only the fully fed mosquitoes were maintained on 10% sugar solution soaked in cotton pads for egg laying in the adult insectary and further experimentation(Figure in S1 Fig).

### Fecundity

Three days after blood feeding during the first gonotrophic phase [25], a group of 200 gravid mosquitoes were selected from each blood meal. In four replicates of 50 mosquitoes each, every mosquito was individually provided with oviposition cups lined with moist filter papers to lay eggs separately. The gravid mosquitoes were allowed to oviposit on the lined substrates. The eggs laid on the filter papers were carefully removed from each oviposition cups using the forceps. The eggs were counted under a dissecting microscope (Olympus CX21FS1) to establish fecundity. Fecundity determines the reproduction rate of an organism and is characterized by the number of progenies an individual mosquito can produce.

### Hatchability

The eggs were incubated for 24 hours to enhance optimal hatchability (the proportion of eggs able to survive through embryonic development to give forth larvae). Each batch of eggs was counted separately and placed in individual trays containing spring water. The trays were lined with filter papers to confine the floating eggs and prevent desiccation. The larvae (L_1_) were allowed to hatch from the eggs after 24 hours. The hatched larvae (L_1_) were counted and placed in larval trays to determine hatchability. The hatchability of the eggs was established by counting the neonate larvae that emerged from the batch of eggs from each mosquito.

### Larval/pupal survivorship

For each blood meal host, a total of 2800 larvae (L_1_) in four replicates of 700 were monitored for survivorship (the proportion of larvae alive at a given period after emergence to pupation or death), pupation time (the mean number of days taken by each larvae to pupate from the start of larval cohort to the end in each blood meal), and pupation rate (the proportion of larvae that successfully pupated in each blood meal).The larvae were kept in standard larval trays of 20x10x8cm with a water depth of 4 cm each accommodating 100 larvae. The larvae were maintained with insectary food, Tetramin (Tetramin Tropical Flakes-Spectrum Brands, Inc, Melle, Germany) provided daily in the morning and in the evening at the rate of 0.003 g/mosquito larva. Rearing water was changed after every two days, trays cleaned, and sun dried. The larval survivorship was monitored by recording mortality and pupae development. The subsequent pupae development was monitored by picking daily the developed pupae using a Pasteur pipette.

### Adult survivorship

The collected pupae were transferred to the adult insectary and placed in the rearing cages measuring 30 cm x 30 cm. Pupae were allowed to emerge into adult mosquitoes and were provided with 10% sucrose solution soaked in cotton pads placed on top of the cages. Upon emergence, 200 female and male adult mosquitoes in 2 replicates of 100 mosquitoes each from every bloodmeal were monitored for adult survival rate (the proportion of adult mosquitoes alive at a given period after emergence to death). The sex ratio and evaluation of subsequent generations were not monitored hence further research is needed to establish this gap in knowledge. Monitoring for survivorship was from the day of emergence (day 0) to the last day the mosquitoes from each blood meal died. The adults were counted daily to establish alive from the dead ones and the results were scored until the last one in each blood meal cage died.

### Data analysis

The differences in feeding success, fecundity, females that laid egg, egg hatchability, pupation rate and time, larval, pupal and adult survival rates were analyzed using one way Analysis of Variance (ANOVA). A *t*-test was performed to compare the statistical significance of variance between the blood hosts and a post hoc (Bonferroni adjustment) done to compare each blood host with human blood host. Feeding success was calculated as the number of mosquitoes that successfully fed (fully fed) divided by the total number of mosquitoes that were exposed to feed multiplied by 100 in each blood type. Fecundity was calculated as the total number of eggs laid by each mosquito in each bloodmeal divided by the total number of eggs laid in a single bloodmeal. Females that laid eggs was determined by finding the mean of the females that laid in each blood host. Hatchability was calculated as the number of larvae that hatched per individual mosquito divided by the number of eggs per individual mosquito multiplied by 100 in each blood host. The effect of bloodmeal on survival of *An. gambiae* s.s. larvae and adults were calculated using Kaplan-Meier survival analysis and curves plotted in excel. Statistical analysis was done using Version 14 of statistical program Stata, StataCorp, College Station, Texas.

Kaplan Meier survival rates.


$$P_{j}=\frac{nj-dj}{nj}$$

Where: *P*_*j*_ = Estimated survival probability or rate of alive mosquitoes at the initial time.

*d*_*j*_ = Number of mosquitoes that died in the same bloodmeal group at the initial time.

*n*_*j*_ = Number of mosquitoes in each bloodmeal monitored for survivorship at initial time.

Survival distribution S(t).


$$\text{S(t)}=\prod_{j:t\;j\leq t}\left(1-\frac{dj}{nj}\right)$$

Where: П =  Cumulative survival product of mosquitoes that survived up to time all the monitored mosquitoes in each bloodmeal died.

tj = Ordered time that at least mosquitoes from each bloodmeal died.

*d*_*j*_ = Number of mosquitoes that died in the same bloodmeal group at the initial time.

*t* = Survival time of mosquitoes in each bloodmeal until dead.

### Ethics approval and consent to participate

The study approval was provided by the Kenya Medical Research Institute/ Scientific and Ethics Review Unit (KEMRI/SERU 3434). There were no human subjects that were used to acquire human blood therefore no consent was sought. The human blood used in this study was obtained from local blood bank. The blood was not infectious but unsuitable for transfusion. All animals blood were collected from the local slaughter houses therefore no animals were directly involved in this work.

## Results

### Feeding success

A total of 3360 female mosquitoes fed on the six different host blood sources (human, cow, goat, pig, chicken, and sheep) in four replicates of 140 mosquitoes for each host. Comparison between human blood and other bloodmeals showed a significant difference in the mean feeding success (F_5,18_ =  35.34, *p* < .001; Table 1). Of the host bloodmeals that were investigated, human bloodmeal had the highest mean in feeding success 125.25 (n = 501) though with no significant difference with pig bloodmeal (*t* (6) = 0.453, *p* =  0.666), while goat bloodmeal 63.75 (n = 255) had the lowest feeding success.

**Table 1:** The average feeding success, the proportion of the female mosquitoes that successfully laid eggs and mean fecundity (number of eggs laid per mosquito in each host blood) of *An. gambiae* after blood feeding on each bloodmeal host.

Host (blood	Feeding success		Females that laid egg		Fecundity (eggs laid)	
meal)	Mean (n)	Post hoc	Mean ± SD	Post hoc	Mean ± SE	Post Hoc
		*p* = 0.010		*P* = 0.010		*P* = 0.010
Sheep	105.00 (420) ^a^	0.024	23.25 ± 2.62	<.001	50.03 ± 7.99	<.001
Goat	63.75 (255)	<.001	16.00 ± 4.08	<.001	45.63 ± 2.81	<.001
Cow	84.50 (338)	<.001	21.50 ± 4.93	<.001	52.28 ± 11.14	<.001
Chicken	108.00 (432)	<.001	27.50 ± 5.44 ^a^	0.021471	63.10 ± 17.14	0.00171
Pig	123.25 (493) ^a^	0.666	26.25 ± 2.87	0.001173	100.46 ± 6.36^a^	0.040114
Human	125.25 (501)	***	36.50 ± 2.08	***	111.65 ± 5.74	***

*Post hoc p = 0.010 (Bonferroni adjustment), only one gonotrophic cycle was monitored in this experiment,*

*a = no significant difference with human bloodmeal;*

***= Comparator.

### Fecundity based on bloodmeal source

A total of 39233 eggs were laid during the entire study from different host bloodmeals. The mean number of females that laid eggs successfully varied significantly between different bloodmeals (*F*_5,18_ = 12.57, *p* < .001). Human bloodmeal had the highest mean of females that laid eggs (mean 36.50). However, chicken bloodmeal (mean =  27.50, *P* = 0.021) did not show any significant difference with human bloodmeal (t (6) = 3.0869, *p* = 0.0214; Table 1. The mean number of eggs laid per mosquito in each bloodmeal was significantly different across different bloodmeals (F_5,18_ = 34.23, *p* < .001). Human bloodmeal (mean =  111.65) had the highest mean number of eggs laid across the bloodmeal sources. However, there was no statistical difference between human bloodmeal and pig bloodmeals (*t* (6) =  2.6101, *p* =  0.04011; Table 1).

### Hatchability, pupation time and pupation rate

Eggs laid from 20 female mosquitoes from each bloodmeal was monitored for hatching rate across the bloodmeal source. There was a significant difference in hatchability (*F*_5, 1114_ =  37.63, *p* < .001) between host bloodmeal sources. Eggs derived from human bloodmeal (88.35%) had the highest hatchability compared to the other bloodmeals investigated. However, there was no significant difference between human and pig bloodmeal *t* (38) =  0.52857, *p* =  0.60017; Table 2. The pupation time (*F*_5,18_ = 5.53, *p* < .001) also significantly varied across bloodmeal sources. The larvae from mosquitoes fed on sheep blood exhibited the longest pupation time (11.39 days), while those fed on goat blood recorded the shortest pupation time (9.79 days). Pupation rate (*F*_5,18_ = 8.26, *p* < .001) likewise varied significantly across the bloodmeals with pig bloodmeal recording the highest (83.50%), and this did not exhibit any significant difference from the pupation rate observed with human bloodmeal (*t* (6) -0.1574, *p* = 0.8800); Table 2).

**Table 2:** Comparisons of the hatching rate, pupation time, and pupation rate of *An. gambiae* across different bloodmeals in contrast to the human bloodmeal.

Host (blood type)	Hatching rates (%)	Post hoc P = 0.010	Pupation time (Days) (Mean ± SD)	Post hoc (P = 0.010)	Pupation rate (%)	Post hoc P = 0.010
Sheep	71.2	>.001	11.39 ± 0.4	>.001	71.5 ± 5.8	0.010
Goat	64.5	>.001	9.79 ± 0.4	0.094^a^	67.5 ± 4.8	0.003
Cow	79	>.001	10.89 ± 0.8	0.244^a^	77 ± 2.2	0.003
Chicken	81.3	>.001	10.19 ± 0.5	0.649^a^	72.8 ± 4.4	0.001
Pig	87.6	0.600^a^	10.62 ± 0.3	0.240^a^	83.5 ± 4.8	0.880^a^
Human	88.4	***	10.33 ± 0.4	***	83 ± 4.2	***

Post hoc p = 0.010 (Bonferroni adjustment), a =  no significant difference with human bloodmeal; *** =  Comparator.

### Larval survivorship

There was no notable variance in the average survival rate of larvae resulting from the different bloodmeal sources examined (*F*_5,92_ =  0.13, *p* = 0.985); Table 3). However, a slightly higher survival rate was evident for pig bloodmeal (mean =  0.57) in comparison to human bloodmeal (mean =  0.55) as well as all the other bloodmeal sources; Table 3.

**Table 3:** Comparison of larval and adult mean survival rates of *An. gambiae s.s.* across different bloodmeals in relation to human bloodmeal*.*

Host blood meal	Larval survivorship			Adult survivorship			
	mean SR (SE)	t-test *p* = value	Median SR (SE)	Mean SR (SE)	t-test *p* = value	Median SR (SE)	Mean days.
Sheep	0.53 ± 0.01^a^	0.889431	0.67 ± 0.43	0.57 ± 0.05	0.020335	0.67 ± 0.36	23
Goat	0.46 ± 0.10 ^a^	0.534335	0.53 ± 0.41	0.64 ± 0.05^a^	0.176861	0.86 ± 0.37	22
Cow	0.52 ± 0.10 ^a^	0.836625	0.72 ± 0.42	0.48 ± 0.06^a^	0.001364	0.52 ± 0.42	19
Chicken	0.54 ± 0.11^a^	0.930866	0.80 ± 0.42	0.66 ± 0.05^a^	0.26783	0.75 ± 0.35	24
Pig	0.57 ± 0.11^a^	0.918919	0.84 ± 0.45	0.67 ± 0.04^a^	0.281971	0.77 ± 0.32	28^a^
Human	0.55 ± 0.11	***	0.83 ± 0.45	0.73 ± 0.04	***	0.92 ± 0.34	28^a^

*SR =  survival rate*

a = no significant difference with human bloodmeal; *** = Comparator.

### Adult survivorship

**Fig 1:**
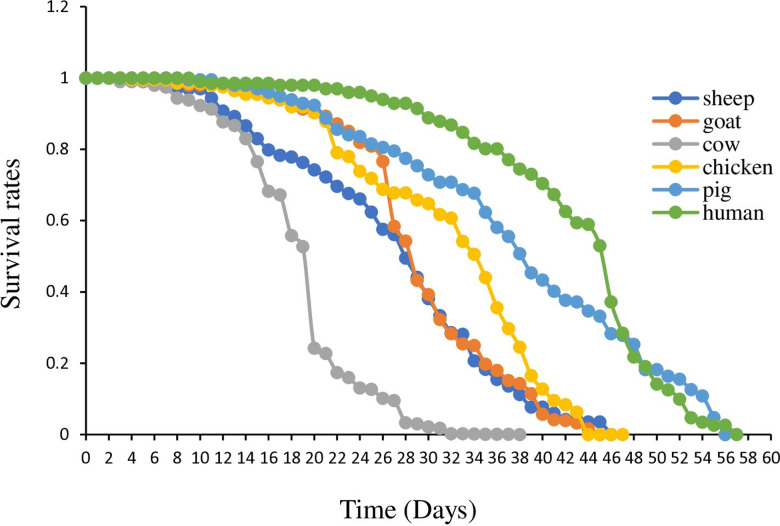
Adult survival rates and longevity of *An. gambiae* s.s. F1 on different host blood meals.

Overall, the average survival rates of first filial generation (F_1_) adults, whose F_0_ (parent) fed on different bloodmeals varied significantly across different bloodmeal sources (*F*_6,346_ = 133.19, *p* < .001); Table 3. Human bloodmeal had the highest mean survival rate (0.73) compared with the rest of the bloodmeals; however, this did not show a significant difference with pig blood *t* (113) = 1.0810, *p* = 0.2819, chicken blood *t* (104) =  1.1140, *p* = 0.2678) and goa*t* blood *t* (102) = 1.3598, *p* = 0.1768; Table 3. A*t* median survival rate, adults resulting from goat bloodmeal (0.86; Table 3) had the highest mean of adults surviving. However, in terms of longevity, pig bloodmeal (56 days) demonstrated the longest survival period, closely rivaling that of human bloodmeal (57 days) (Fig 1) with equal mean survival days (28); Table 3.

## Discussion

Our results indicated that bloodmeal derived from pig performed just as well as bloodmeal derived from humans across the indicators evaluated. In addition, no significant statistical difference was observed between bloodmeals derived from pig and humans, depicting it as a possible alternative to human bloodmeal for mass rearing of *An. gambiae* mosquitoes. Mosquitoes fed on pig bloodmeals had comparable results to those that fed on humans which suggests that pigs are as good a host as humans for this vector species and may be an excellent blood source for mass rearing of mosquitoes. Similarities in biochemical and physiological factors in human and pig blood such as immunological responses, amino acid levels and RBCs components denote that the two hosts can perform comparable biological functions in *Anopheles* mosquitoes mass production. [26]. The pig red blood cells (RBCs) have a diameter of 4-8 µm and the RBCs for humans has a diameter of 6-8 µm showing a similar RBCs average size in both blood host which may enable them to function equally in oxygen carrying capacity [27]. The relatively similar RBC count for human (5.7 – 6.9 million/ µ L) and pig (4.2 – 6.2 million/ µ L) could mean that pig bloodmeal can supplement similar quantity advantage of nutrients as from human [27]. The overlying tendency in hemoglobin levels between human blood (12 – 18 g/100ml) and pig blood (6 – 18 g/100ml) may suggest an equivalent yield of necessary nutrients for vitellogenesis and egg production in *Anopheles* mosquitoes [27]. Hemoglobin, a very crucial blood component, provides critical irons and protein requirements for the mosquito’s reproductive process such as signaling pathways for vitellogenesis, where vitellin binds to 30 or more heme molecules and facilitate embryo development [28–30]. Our results displayed no significant difference between the two bloodmeals which postulate equal support in egg production for bloodmeals from humans and pigs. The minor difference in isotonicity (NaCl concentration) between pig (0.85%) and human (0.9%) blood infers an almost equal osmotic pressure in both bloodmeals [27] which will maintain essential biochemical functions efficiently [2]. The similar total RBCs volumes in pig (56-95 ml/kg) and humans (65-75 mg/kg) suggest that *Anopheles* mosquitoes can extract comparable amounts of nutrients from either source [26]. The quantities of amino acid in pigs and humans blood are much more similar [31]. Isoleucine (3.0g aa/100g aa) in both human and pig blood while phenylalamine (3.9 and 4.0g aa/100 aa) in human and pig respectively are important in energy production, oogenesis and egg laying, blood digestion, and tissue repair [32,33]. Comparing the immune system function of pigs and humans, the closely undistinguishable immunological response are over 80% which include the immune proteins such as complement proteins, cytokines, and antibodies [34,35]. This provides a similar immunological environment and responses in pig bloodmeal as of human bloodmeal. Both pigs and human blood present complexity in blood antigen diversity with complex blood group systems [36]. However, it is confirmed that pig blood contain blood group antigens A-O(H) system that slackly resemble the human ABO blood group system, with a DNA homology with human ABO murine genes, permitting for being classified to the A,B, and O groups in human [37]. Pig blood biochemical and physiological properties are much more like human which can permit xenotransfusion [26] further supporting the study findings. In addition, in many countries, pigs are domestically kept as a source of pork and therefore their blood is widely available and may be cheap to find in local abattoirs where animal rights, welfare, and policies are regulated and adhered to. As with human blood, standard precautions must be taken into consideration when handling pig blood meal before feeding and during disposal after every feeding session.

With the rising interest in genetically modified mosquitoes for vector control, there is a growing demand for improved rearing practices for mass production. Therefore, blood feeding of reared mosquitoes forms the basis of a successful mass production of mosquitoes. Human blood meal performed best as was expected, given *An. gambiae* s.s. anthropophilic tendencies [38]. The precise reason why pig and human blood were superior to other meal types were not investigated and remains unknown to this study. However, Mamai *et al* [3] attributes this variation to the differences in hematological properties across the animal kingdom. Identifying a nutrient-rich blood meal for vitellogenesis is crucial to maximize egg production, leading to an increased overall output to meet substantial demands for mass rearing production [3]. Although the proportion of females that laid eggs and fecundity remained highest in mosquitoes fed on human blood meal, pig blood meal produced statistically comparable outcomes. This denotes that pig blood meal can equally supplement required nutritional values as human blood meal for egg production. Similar scenarios were noted [39] when *Glossina morsitans* fed on pig blood meal and [40], *Aedes albopictus* fed on swine blood meal compared to other blood meal sources. Of the blood meal hosts evaluated in this study, chicken blood meal performance was best in females that were able to lay eggs and pupation time. This performance may be attributed to the physiology and biochemistry of its oval and nucleated erythrocytes with a shorter lifespan of 35 days only, in supporting the tested parameters [41]. Overall, the sheep and cow blood meals exhibited poor results in all the parameters investigated from the rest of the blood meal hosts. Our results showing poor performance of sheep blood meal are consistent with [4] and [17] outcomes when *Anopheles* and *Aedes* mosquitoes displayed very low fecundity and hatching rates after being fed on sheep blood meal. Though a few studies found cow blood meal to be the best in rearing of *Anopheles* mosquitoes, pig blood meal was not evaluated [42]. The poorest performed blood meal was from the goat with very low feeding success, fewest number of eggs laid and females that laid eggs, low hatching and pupation rates, shortest larval and adult survivorship. However, the goat blood meal performed best in pupation time resulting in the shortest time to complete the life cycle for mass production. From our understanding, goat blood meals have not been evaluated broadly in blood feeding of *Anopheles.* Our evaluation majorly focused on fitness outcomes: feeding success, fecundity, hatchability and larval and adult survivorship. Other fitness characterization factors such as body size and mating success were not evaluated, and this was a limitation to this study.

These results are important for adoption in mass rearing and production with benefits; (i) avert the consequences that come with direct live feeding on animals such as ethical concerns and animal rights. (ii) It’s satisfying that *An. gambiae* can reproduce successfully with equal quality output by utilizing an alternative blood meal through artificial membrane from other blood sources other than human host. *An. gambiae* mosquitoes require repetitive blood meals to enable egg formation [43]. However, this was not the case in this study since mosquitoes were allowed to take only a single blood meal. This was a limitation on the outcome as we do not know how the results from repetitive feeding per blood meal type would present on all the measured parameters, and this calls for more investigation regarding repetitive blood feeding.

## Conclusion

Results from this study demonstrate the potential of pig blood being an alternative blood source in the mass production for *An. gambiae*. Blood feeding, fecundity and adult survivorship outcomes were similar between pig and human blood meals. The documented biochemical and physiological similarities between human and pig blood - such as red blood cell count, red blood cell size, isotonicity, hematocrit, hemoglobin levels, immunological response and amino acid composition denote that these blood hosts perform comparable biological functions. This implies that *Anopheles* mosquitoes feeding on pig blood obtain similar amount of nutrients and oxygen carrying capacity as they would from human blood. The overlapping level of hemoglobin in pig and human blood signifies that mosquitoes can obtain sufficient nutrients to support crucial reproductive processes. Furthermore, the quantity resemblance in some amino acid such as isoleucine and phenylalamine which are responsible regulation of in egg laying behavior, oogenesis, blood digestion, and tissue repair. With over 80% similarity in immune system function, pig and human blood present mosquitoes to a closely undistinguishable immunological response. Moreover, antigenic similarities between these two blood hosts, the pig A-O(H) blood group system, and the human ABO blood system, further underlines the insignificance in the results from both hosts. However, pig bloodmeals have not been largely investigated on mass rearing and production hence more research is required.

## Supporting information

S1 Fig Flow chart showing experimental set up. (TIF)

S1 Table Host identification, blood collection and storage (DOCX)

## Abbreviations

LLIN: Long-Lasting Insecticidal Treated Nets
IRS: Indoor Residual Spraying
WHO: World Health Organization
WMR: World Malaria Report
CRISPR: Clustered Regulatory Interspaced Short Palindromic Repeats
PGSIT: Precision-Guided Sterile Insect Technique
GRSIT: Gamma-Radiated Sterile Insect Technology
DLG: Dominant Lethal Gene
CAS9: CRISPR Associated Protein 9
fsRIDL: Female-Specific Release of Insects carrying a Dominant Lethal
KEMRI: Kenya Medical Research Institute
CGHR: Centre for Global Health Research
EDTA: Ethylenediamine Tetra Acetic Acid
UK: United Kingdom
L_1_: Larval Stage One
ANOVA: Analysis of Variance
*An.*: *Anopheles*
s.s.: Sensu Stricto
n: Sample Size
P: Probability
SE: Standard Error
SD: Standard Deviation
SR: Survival Rate.

## References

[1] Sinka ME, Bangs MJ, Manguin S, Coetzee M, Mbogo CM, Hemingway J, et al. The dominant Anopheles vectors of human malaria in Africa, Europe and the Middle East: occurrence data, distribution maps and bionomic précis. Parasit Vectors. 2010;3 117. doi: 10.1186/1756-3305-3-117 21129198 PMC3016360

[2] Clements AN. The Biology of Mosquitoes: Development, Nutrition, and Reproduction. CABI Publishing 1999;Volume 1.

[3] Mamai W, Bimbile-Somda NS, Maiga H, Juarez JG, Muosa ZAI, Ali AB, et al. Optimization of mosquito egg production under mass rearing setting: effects of cage volume, blood meal source and adult population density for the malaria vector, Anopheles arabiensis. Malar J. 2017;16(1):41. doi: 10.1186/s12936-017-1685-3 28118825 PMC5260048

[4] Phasomkusolsil S, Tawong J, Monkanna N, Pantuwatana K, Damdangdee N, Khongtak W, et al. Maintenance of mosquito vectors: effects of blood source on feeding, survival, fecundity, and egg hatching rates. J Vector Ecol. 2013;38(1):38–45. doi: 10.1111/j.1948-7134.2013.12006.x 23701605

[5] Tandina F, Doumbo O, Yaro AS, Traoré SF, Parola P, Robert V. Mosquitoes (Diptera: Culicidae) and mosquito-borne diseases in Mali, West Africa. Parasit Vectors. 2018;11(1):467. doi: 10.1186/s13071-018-3045-8 30103823 PMC6090629

[6] World Health Organization. Seventh meeting of the vector control advisory group, Geneva, Switzerland, 24-26 October 2017. Geneva: World Health Organization; 2017. (WHO/HTM/NTD/VEM/2017.11). Licence: CC BY-NC-SA 3.0 IGO.

[7] Sun D, Guo Z, Liu Y, Zhang Y. Progress and prospects of CRISPR/Cas systems in insects and other arthropods. Frontiers in Physiology. 2017;8(1):1–10. doi: 10.3389/fphys.2017.00001 28932198 PMC5592444

[8] Li M, Yang T, Bui M, Gamez S, Wise T, Kandul NP, et al. Suppressing mosquito populations with precision guided sterile males. Nat Commun. 2021;12(1):5374. doi: 10.1038/s41467-021-25421-w 34508072 PMC8433431

[9] Gentile JE, Rund SSC, Madey GR. Modelling sterile insect technique to control the population of Anopheles gambiae. Malar J. 2015;14:92. doi: 10.1186/s12936-015-0587-5 25889145 PMC4351850

[10] Fu G, Lees RS, Nimmo D, Aw D, Jin L, Gray P, et al. Female-specific flightless phenotype for mosquito control. Proc Natl Acad Sci U S A. 2010;107(10):4550–4. doi: 10.1073/pnas.1000251107 20176967 PMC2826341

[11] Labbé GMC, Scaife S, Morgan SA, Curtis ZH, Alphey L. Female-specific flightless (fsRIDL) phenotype for control of Aedes albopictus. PLoS Negl Trop Dis. 2012;6(7):e1724. doi: 10.1371/journal.pntd.0001724 22802980 PMC3393675

[12] Wang G-H, Gamez S, Raban RR, Marshall JM, Alphey L, Li M, et al. Combating mosquito-borne diseases using genetic control technologies. Nat Commun. 2021;12(1):4388. doi: 10.1038/s41467-021-24654-z 34282149 PMC8290041

[13] Carvalho DO, McKemey AR, Garziera L, Lacroix R, Donnelly CA, Alphey L, et al. Suppression of a field population of aedes aegypti in brazil by sustained release of transgenic male mosquitoes. PLoS Negl Trop Dis. 2015;9(7):e0003864. doi: 10.1371/journal.pntd.0003864 26135160 PMC4489809

[14] Paes de Andrade P, Aragão FJL, Colli W, Dellagostin OA, Finardi-Filho F, Hirata MH, et al. Use of transgenic Aedes aegypti in Brazil: risk perception and assessment. Bull World Health Organ. 2016;94(10):766–71. doi: 10.2471/BLT.16.173377 27843167 PMC5043214

[15] Oxitec. Government of djibouti and oxitec announce importation of friendly^TM^ anopheles stephensi mosquitoes. oxitec 2024. [cited 2024 March 20] Available from: https://www.oxitec.com/en/news/government-of-djibouti-and-oxitec-announce-importation-of-friendly-anopheles-stephensi-mosquitoes

[16] Roitberg BD, Gordon I. Does the Anopheles blood meal-fecundity curve, curve?. J Vector Ecol. 2005;30(1):83–6. 16007959

[17] Shehu S, Muhammad Sulaiman H. Effect of Different Blood Meal on Feeding Rate and Fecundity of Aedes and AnophelesSpp (Diptera: Culicidae). 2019. https://www.researchgate.net/publication/338105114_Effect_of_Different_Blood_Meal_on_Feeding_Rate_and_Fecundity_of_Aedes_and_AnophelesSpp_Diptera_Culicidae

[18] Dias LDS, Bauzer LGS da R, Lima JBP. Artificial blood feeding for Culicidae colony maintenance in laboratories: does the blood source condition matter?. Rev Inst Med Trop Sao Paulo. 2018;60 e45. doi: 10.1590/s1678-9946201860045 30231167 PMC6169092

[19] Hasyim H, Dhimal M, Bauer J, Montag D, Groneberg DA, Kuch U, et al. Does livestock protect from malaria or facilitate malaria prevalence? A cross-sectional study in endemic rural areas of Indonesia. Malar J. 2018;17(1):302. doi: 10.1186/s12936-018-2447-6 30126462 PMC6102806

[20] Olayemi IK, Ande AT, Danlami G, Abdullahi U. Influence of blood meal type on reproductive performance of the malaria vector, anopheles gambiae s.s. (Diptera: Culicidae). J of Entomology. 2011;8(5):459–67. doi: 10.3923/je.2011.459.467

[21] Gunathilaka N, Ranathunge T, Udayanga L, Abeyewickreme W. Efficacy of blood sources and artificial blood feeding methods in rearing of aedes aegypti (Diptera: Culicidae) for sterile insect technique and incompatible insect technique approaches in Sri Lanka. Biomed Res Int. 2017;20173196924. doi: 10.1155/2017/3196924 28894749 PMC5574269

[22] Machani MG, Ochomo E, Sang D, Bonizzoni M, Zhou G, Githeko AK, et al. Influence of blood meal and age of mosquitoes on susceptibility to pyrethroids in Anopheles gambiae from Western Kenya. Malar J. 2019;18(1):112. doi: 10.1186/s12936-019-2746-6 30940139 PMC6444593

[23] Ndenga BA, Mulaya NL, Musaki SK, Shiroko JN, Dongus S, Fillinger U. Malaria vectors and their blood-meal sources in an area of high bed net ownership in the western Kenya highlands. Malar J. 2016;15 76. doi: 10.1186/s12936-016-1115-y 26857915 PMC4746809

[24] Banfi G, Salvagno GL, Lippi G. The role of ethylenediamine tetraacetic acid (EDTA) as in vitro anticoagulant for diagnostic purposes. Clin Chem Lab Med. 2007;45(5):565–76. doi: 10.1515/CCLM.2007.110 17484616

[25] Briegel H, Gut T, Lea AO. Sequential deposition of yolk components during oogenesis in an insect, Aedes aegypti (Diptera: Culicidae). J Insect Physiol. 2003;49(3):249–60. doi: 10.1016/s0022-1910(02)00272-x 12770000

[26] Smood B, Hara H, Schoel LJ, Cooper DKC. Genetically-engineered pigs as sources for clinical red blood cell transfusion: What pathobiological barriers need to be overcome?. Blood Rev. 2019;35:7–17. doi: 10.1016/j.blre.2019.01.003 30711308 PMC6467751

[27] Long C, Hara H, Pawlikowski Z, Koike N, d’Arville T, Yeh P, et al. Genetically engineered pig red blood cells for clinical transfusion: initial in vitro studies. Transfusion. 2009;49(11):2418–29. doi: 10.1111/j.1537-2995.2009.02306.x 19624491

[28] Logullo C, Moraes J, Dansa-Petretski M, Vaz IS, Masuda A, Sorgine MHF, et al. Binding and storage of heme by vitellin from the cattle tick, Boophilus microplus. Insect Biochem Mol Biol. 2002;32(12):1805–11. doi: 10.1016/s0965-1748(02)00162-5 12429132

[29] Walter-Nuno AB, Oliveira MP, Oliveira MF, Gonçalves RL, Ramos IB, Koerich LB, et al. Silencing of maternal heme-binding protein causes embryonic mitochondrial dysfunction and impairs embryogenesis in the blood sucking insect Rhodnius prolixus. J Biol Chem. 2013;288(41):29323–32. doi: 10.1074/jbc.M113.504985 23986441 PMC3795234

[30] Perner J, Sobotka R, Sima R, Konvickova J, Sojka D, Oliveira PL de, et al. Acquisition of exogenous haem is essential for tick reproduction. Elife. 2016;5 e12318. doi: 10.7554/eLife.12318 26949258 PMC4821805

[31] Miller ER, Ullrey DE. The pig as a model for human nutrition. Annu Rev Nutr. 1987;7:361–82. doi: 10.1146/annurev.nu.07.070187.002045 3300739

[32] Wu G, Ott TL, Knabe DA, Bazer FW. Amino Acid Composition of the Fetal Pig. J Nutr. 1999;129(5):1031–8. doi: 10.1093/jn/129.5.1031 10222396

[33] Marquardt WC, Kondratieff BC. Biology of Disease Vectors. Elsevier Science;pp. 785. Elsevier Academic Press, USA,2005.ISBN0 12 473276. doi:doi: 10.1017/S0031182005219674

[34] Dawson H. A comparative assessment of the pig, mouse and human genomes: Structural and functional analysis of genes involved in immunity and inflammation. In: McAnulty PA, Dayan A, Hastings KH, Ganderup N-C, editors. The minipig in Bbomedical research. Boca Raton, FL: CRC Press; 2011. p. 321–41.

[35] Fairbairn L, Kapetanovic R, Sester DP, Hume DA. The mononuclear phagocyte system of the pig as a model for understanding human innate immunity and disease. J Leukoc Biol. 2011;89(6):855–71. doi: 10.1189/jlb.1110607 21233410

[36] Meurens F, Summerfield A, Nauwynck H, Saif L, Gerdts V. The pig: a model for human infectious diseases. Trends Microbiol. 2012;20(1):50–7. doi: 10.1016/j.tim.2011.11.002 22153753 PMC7173122

[37] Yamamoto F, Yamamoto M. Molecular genetic basis of porcine histo-blood group AO system. Blood. 2001;97(10):3308–10. doi: 10.1182/blood.v97.10.3308 11342465

[38] Basseri H, Raeisi A, Ranjbar Khakha M, Pakarai A, Abdolghafar H. Seasonal abundance and host-feeding patterns of anopheline vectors in malaria endemic area of iran. J Parasitol Res. 2010;2010671291. doi: 10.1155/2010/671291 21559055 PMC2943101

[39] Mews AR, Langley PA, Pimley RW, FLOOD MET. Large-scale rearing of tsetse flies (Glossina spp.) in the absence of a living host. Bull Entomol Res. 1977;67(1):119–28. doi: 10.1017/s0007485300010944

[40] Hwang S, Kim D. Optimization of artificial feeding system for mass rearing of the Asian tiger mosquito, Aedes albopictus. Entomological Research. 2021;51(11):543–51. doi: 10.1111/1748-5967.12547

[41] el-Mekawi S, Yagil R, Meyerstein N. Effect of oxidative stress on avian erythrocytes. J Basic Clin Physiol Pharmacol. 1993;4(3):199–211. doi: 10.1515/jbcpp.1993.4.3.199 8679516

[42] Chikwendu J, Onekutu A, Ogbonna I. Effects of Host Blood on Fecundity and Longevity of Female Anopheles Mosquitoes. Int J Pathog Res. 2019:1–7. doi: 10.9734/ijpr/2019/v3i230091

[43] Gillies MT. The recognition of age-groups within populations of Anopheles gambiae by the pre-gravid rate and the sporozoite rate. Ann Trop Med Parasitol. 1954;48(1):58–74. doi: 10.1080/00034983.1954.11685599 13149120

