# Supplementary figures and images for: Influence of different host blood meal sources on the reproductive outcomes in Anopheles gambiae: Enhancing fecundity in a mass rearing environment
Source: PLoS One. 2025 Mar 10;20(3):e0307789. doi: 10.1371/journal.pone.0307789 (PMC11892840; doi:10.1371/journal.pone.0307789)

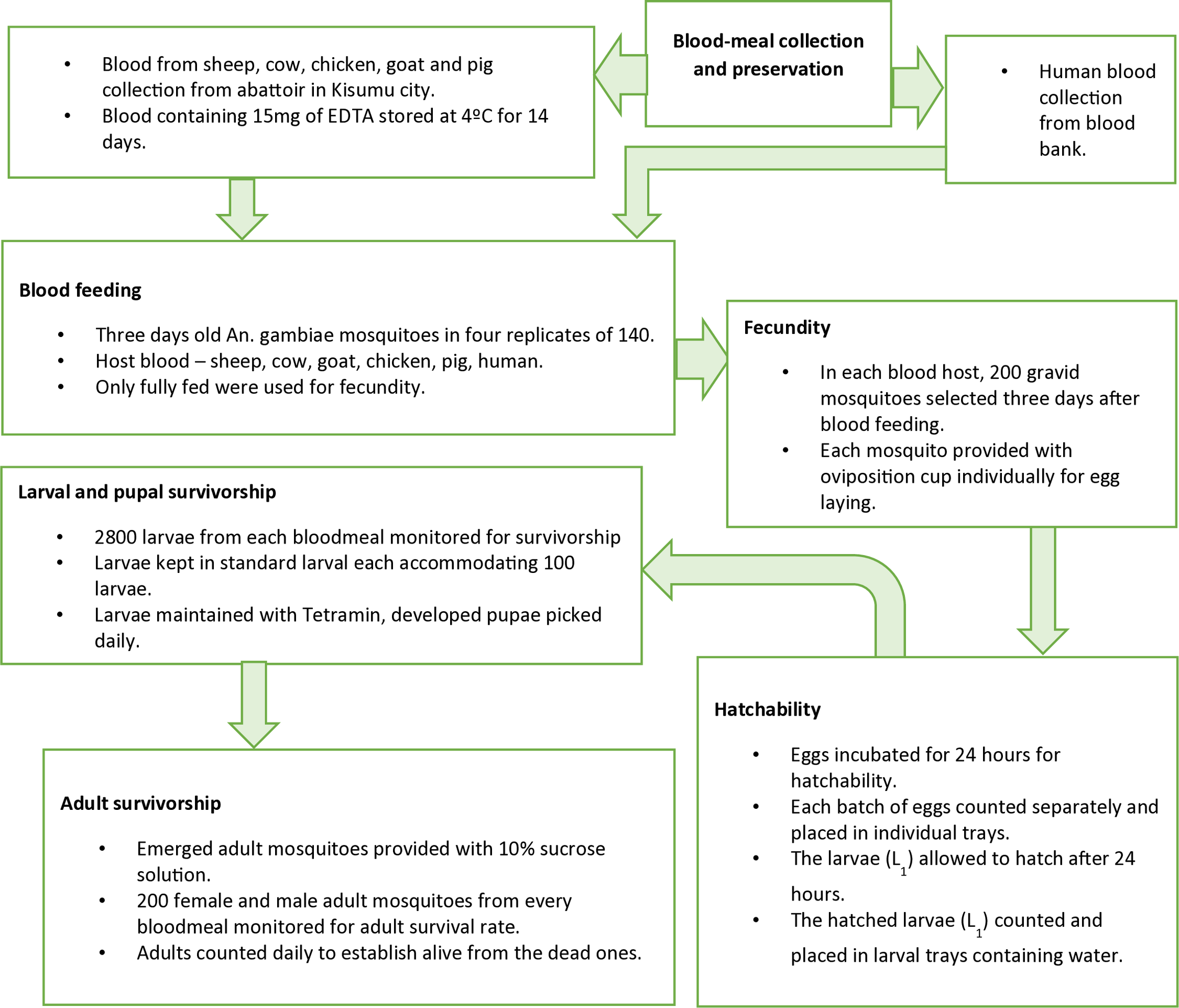

Supplement: S1 Fig — (TIF) [file pone.0307789.s001.tif]
