# Supplementary material for: Influence of different host blood meal sources on the reproductive outcomes in Anopheles gambiae: Enhancing fecundity in a mass rearing environment
Source: PLoS One. 2025 Mar 10;20(3):e0307789. doi: 10.1371/journal.pone.0307789 (PMC11892840; doi:10.1371/journal.pone.0307789)
Supplement: S1 Table — (DOCX) [file pone.0307789.s002.docx]

**Influence of Different Host Blood Meal Sources on the Reproductive Outcomes in *Anopheles gambiae*: Enhancing Fecundity in a Mass Rearing Environment**

Supplementary Table 1: Host identification, blood collection and storage.

| **Animal**  **name** | **Animal**  **species** | **Time of**  **collection** | **Volume of blood**  **collected** | **Preservation method** | **Anticoagulation** |
| --- | --- | --- | --- | --- | --- |
| **Sheep** | *Ovis aries* | 6 am | 50 ml | 4°C refrigeration | EDTA |
| **Cow** | *Bos taurus)* | 6 am | 50 ml | 4°C refrigeration | EDTA |
| **Goat** | *Capra aegagrus hircus* | 6 am | 50 ml | 4°C refrigeration | EDTA |
| **Chicken** | *Gallus gallus domesticus* | 6 am | 50 ml | 4°C refrigeration | EDTA |
| **Pig** | *Sus scrofa domesticus* | 6 am | 50 ml | 4°C refrigeration | EDTA |
